# Efficacy of Cefquinome against *Escherichia coli* Environmental Mastitis Assessed by Pharmacokinetic and Pharmacodynamic Integration in Lactating Mouse Model

**DOI:** 10.3389/fmicb.2017.01445

**Published:** 2017-08-02

**Authors:** Yang Yu, Jin-Tao Fang, Jian Sun, Mei Zheng, Qing Zhang, Jie-Shun He, Xiao-Ping Liao, Ya-Hong Liu

**Affiliations:** ^1^Guangdong Provincial Key Laboratory of Veterinary Pharmaceutics, Development and Safety Evaluation, South China Agricultural University Guangzhou, China; ^2^National Risk Assessment Laboratory for Antimicrobial Resistance of Animal Original Bacteria, South China Agricultural University Guangzhou, China; ^3^Laboratory of Veterinary Pharmacology, College of Veterinary Medicine, South China Agricultural University Guangzhou, China

**Keywords:** cefquinome, *Escherichia coli*, environmental mastitis, dosage regimen, PK/PD, mouse model

## Abstract

This work investigates the pharmacodynamic effectiveness of cefquinome against environmental *Escherichia coli* mastitis infection, following an intramammary administration. We established the pharmacokinetic and pharmacodynamic (PK/PD) model in lactating mice. The PK/PD parameters were identified to achieve an antibacterial efficacy as indicated by PD activity, cytokine expression and PK/PD simulation. From our findings, given an 200 μg/gland dose once daily can achieve a considerable therapeutic effectiveness in experimental circumstance.

## Introduction

Dairy food products are staples of recommended healthy diets in modern life, and dairy cow farming is a worldwide business. During past decades, economic losses were huge and long-term due to mastitis in the dairy chain (DeGraves and Fetrow, 1993; Seegers et al., 2003; Wolfova et al., 2006; Sinha et al., 2014; Rollin et al., 2015). Bovine mastitis is a significant disease and may cause economic losses, like higher direct costs, lower production, degraded milk quality, culling of cows, and treatment costs (van Soest et al., 2016). Mastitis eradication is difficult and costly, which means that mastitis will likely persist into the near future (DeGraves and Fetrow, 1993). Acute clinical mastitis caused by environmental organisms is becoming a major challenge for farmers, even in well-managed herds (Shpigel et al., 1997). The immediate surroundings are primary reservoirs for environmental mastitis pathogens. Healthy or uninfected udders could be easily exposed to these pathogens during milking or even the dry periods (Gruet et al., 2001).

*Escherichia coli* is one of the most important pathogens inducing clinical environmental mastitis (Gruet et al., 2001). This organism is ubiquitous in the farm. Healthy animals, even humans, are the carriers of this pathogen. Improper milking procedures, season changing, cow behavior or weakened host immunity can provide opportunities for intramammary infection of *E. coli*. Infection and inflammation of the mammary gland caused by *E. coli* in dairy cows are mainly happened around parturition and during early lactation with striking local and sometimes severe systemic clinical symptoms, including acute swelling of quarters, watery milk, anorexia, fever, even septic shock and recumbent ended by death (Burvenich et al., 2003; Abd-Elrahman, 2013). This so-called environmental type of mastitis may affect productivity of high producing cows in dairy herds (Burvenich et al., 2003). Although some measures for prevention have been addressed, effective therapeutic treatments against acute clinical environmental mastitis are still urgently needed (Shpigel et al., 1997).

In our previous studies, better therapeutic efficacy has been observed given once dosage than dosing fractionation following intramammary administration of cefquinome, a fourth generation cephalosporin that is developed solely for veterinary use (Yu et al., 2016a). The anatomic structure of the blood-milk barrier plays a significant role in drug distribution and has noticeable influence on therapeutic efficacy. We compared PK profiles in blood (Yu et al., 2016a) and mammary gland tissues (Yu et al., 2016b) following intramammary infusion, and we found the mammary gland PKs can reflect drug concentration in udder tissue better and are more suitable for PK/PD modeling than blood PKs. In addition, the ratio of area under the concentration-time curve to the minimal inhibitory concentration (AUC/MIC) was found to be the prospective parameter in PK/PD modeling (Yu et al., 2016b).

In the current study, we aimed to investigate the activity of cefquinome against clinical mastitis *E. coli* strains *in vitro*, and evaluate *in vivo* therapeutic efficacy of mouse *E. coli* mastitis by monitoring bacterial colony counts and the cytokine of tumor necrosis factor -α (TNF-α) and interleukin -2 (IL-2). In addition, PK/PD modeling was analyzed using a sigmoid *E_max_* model following intramammary infusion to obtain the magnitude of PK/PD index.

## Materials and Methods

### Bacterial Strains, Animals and *In Vitro* Antibacterial Effect

*Escherichia coli* 8 strain was isolated from milk samples collected from clinical mastitis cows, and identified by Matrix-Assisted Laser Desorption/Ionization Time of Flight Mass Spectrometry (MALDI-TOF-MS) (Shimadzu-Biotech) (Singhal et al., 2015) and sequence analysis of 16S rRNA using universal primers (16S-F, 5′-AGA GTT TGA TCA TGG CTC-3′; 16S-R, 5′-GGT TAC CTT GTT ACG ACT T-3′). Extended-spectrum β-lactamases (ESBLs) resistance genes of *bla*_CTX_, *bla*_TEM_, and *bla*_SHV_ were tested by previously described PCR protocols (Gröbner et al., 2009; Cullik et al., 2010). The phylogenetic typing was determined by multiplex PCR using primers specific for *chuA*, *yjaA* genes and a gene unrelated to antibiotic resistance *TSPE4.C2* (Clermont et al., 2000). Also, susceptibility to cefquinome was conducted following CLSI guidelines (CLSI, 2013). Luria–Bertani (LB) broth and agar were used for bacterial culture and counting, and the strain was stored in multiple aliquots of LB broth with 20% glycerol at -80°C.

Healthy lactating CD-1 female mice, behaving vivaciously and regularly, were weighed 35 to 45 g (Vital River Laboratories, Beijing, China) and housed in a specific-pathogen-free environment. Experimental procedures with these mice were conducted within 1–2 h after removal of 10–12 day-old offspring. Animal experiments were approved by the Animal Use and Care Committee of South China Agricultural University and the guidelines of American Association for Accreditation of Laboratory Animal Care (AAALAC) were respected or followed during all the *in vivo* procedures (Institute of Laboratory Animal Resources et al., 1996).

Antibacterial effects of cefquinome was investigated by *in vitro* time-killing curves against *E. coli* 8 strain. Three different initial bacterial inocula of 10^6^, 10^7^, and 10^8^ CFU/mL and four drug concentrations of 0.5, 1, 4, and 8 × MIC were tested here. Bacterial counting were performed at 0, 3, 6, 9, and 24 h, respectively. At each time point, 100 μL liquid sample was appropriately diluted and plated on the LB agar, and then cultured at 37°C for 18 h.

### Mouse Mastitis Model Caused by *E. coli*

To evaluate the infective activity of *E. coli* in mammary gland tissue, thirty-six healthy lactating CD-1 mice were infected with *E. coli* strain in the fourth abdominal mammary glands (both left and right ones), which are anatomically separated and easily harvested. Therefore, each gland tissue was considered as an individual sample. The experimental conditions were modified from a previous report (Brouillette and Malouin, 2005). In brief, an overnight culture of *E. coli* 8 strain was diluted to 5,000 CFU/mL and a 100 μL sample was infused into the udder canal through a small cut under a teat using a 32-gauge blunt needle. Four mice were randomly selected and sacrificed by cervical dislocation at 2, 4, 7, 10, 12, 24, 48, 72, and 96 h after inoculation. The sample size is eight for mammary glands from 4 mice at each time point (*n* = 8 for glands). Mammary gland tissues were harvested, homogenized and dilutions in phosphate-buffered saline (PBS) were plated on MacConkey Agar (Guangdong Huankai Microbial Science) for counting bacteria.

Therapeutic intervention experiments with cefquinome lasted 3 days to investigate the antimicrobial effectiveness of this drug. Ten treatment groups (12 mice each) received daily cefquinome doses of 25, 50, 100, 200, and 400 μg/gland at 12 or 24 h intervals by intramammary infusion into both the fourth abdominal glands. Cefquinome was intramammary administrated into the glands at 9 h following inoculation. Both mammary glands and kidneys of four mice were harvested at 24, 48, and 72 h for bacterial counts, and every single gland or kidney was considered as an individual sample (*n* = 8 for gland or kidney at each time point). In the growth control group, sterile saline solution instead of cefquinome was given and the initial bacterial density was determined at 0 h.

After colony count determination, all mammary gland homogenate were centrifuged immediately and supernatants were stored at -20°C for cytokine test within a week.

### Cytokine ELISA

TNF-α, and interleukins 1, 2, 6, and 8 levels were determined from supernatants using commercial ELISA Kits (Mlbio, Shanghai). All samples were analyzed following protocols suggested by the manufacturer in triplicate.

### Pharmacokinetics

A single dose PK study of cefquinome was carried out using 144 individual healthy lactating mice following intramammary administration of 12.5, 50, 200, and 800 μg cefquinome per fourth gland. Mammary gland organs were harvested at 5, 10, 15, 30, 45 min, 1, 2, 3, 4, 8, 12, and 24 h using three mice at each time point.

Cefquinome in gland samples was quantified using high-performance liquid chromatography (HPLC) as previously reported (Sørensen and Snor, 2000; Zhang et al., 2007; Yu et al., 2016b). Briefly, the mammary gland tissue sample extracted by acetonitrile and cleaned using tC_18_ solid-phase extraction (SPE) cartridge (Waters CO., United States). The analytes were eluted with 2 mL acetonitrile and evaporated under a stream of nitrogen at 38 to 40°C. The drug determination was performed by HPLC (Ultimate 3000, Dionex) equipped with a RP18 column (4.6 mm × 150 mm, 5 μm; Waters Co., United States). The injection volume was 50 μl, and column temperature was maintained at 30°C. The mobile phase consisted of acetonitrile and 5 mM ammonium acetate containing 0.1% formic acid (v/v, 13/87) provided as an isocratic elution with a flow rate of 250 μl/min. The total run time was 7 min. The limit of quantification (LOQ) and detection (LOD) were 0.05 and 0.01 μg/mL, respectively.

The mammary gland tissue PK profiles were analyzed using a non-absorption one-compartment model with WinNonlin software (version 5.2.1; Pharsight, United States). PK parameters using this software included half-life of elimination (*T_1/2Kel_*), initial concentration (*C*_0_), and area under the concentration time curve (AUC_0-24_). PK characteristics of multiple dosing regimens were extrapolated from the corresponding single dose PK data.

### PK/PD Integration

The PK/PD parameters of the dosing regimens in PD experiments were calculated from dosages of 25, 50, 100, 200, 400, and 800 μg/gland administrated once or given twice in fractionated doses. The surrogate markers of the ratio of the AUC_0-24_ to the MIC (AUC_0-24_/MIC) of mammary gland tissue were calculated using equation 1:

$$C_{n}=\frac{{FX}_{0}}{V}(\frac{1-e^{-{nK}_{{el}^{\tau }}}}{1-e^{-K_{{el}^{\tau }}}}).e^{-kt}$$

where *C* is the drug concentration, *n* is the dosing time, *F* is the bioavailability, *V* is the apparent volume of distribution, *X*_0_ is the dose of antibiotic, *K_el_* is the elimination half-life, and τ is the dosing interval.

The antimicrobial effect of cefquinome was analyzed applying the sigmoid *E_max_* model of inhibitory effects as previously reported (Zhao et al., 2014). This is defined as

$$E=E_{\max}-\frac{(E_{\max}-E_{0})\times C_{e}^{N}}{{EC}_{50}^{N}+C_{e}^{N}}$$

where *E* is the antibacterial effect measured as the change in log_10_CFU/g gland after 24 h of treatment compared to the initial colony counts; *E_max_* is the Δlog_10_CFU/g gland in the drug-free control sample; *E_0_* is the Δlog_10_CFU_24h_/g gland in the test sample containing cefquinome when the maximum antibacterial effect was achieved; *C_e_* is the PK/PD index of AUC_0-24_/MIC for gland tissue; *EC*_50_ is the value of PK/PD index of drug producing 50% of the maximum antibacterial effect; and *N* is the Hill coefficient that describes the steepness of the concentration-effect curve.

## Results

### Phylogenetic Typing and MIC Determinations

*Escherichia coli* 8 strain was phylogenetic B_1_ type testing by triplex PCR. The presence of antibiotic resistance genes including *bla*_CTX_, *bla*_TEM_, and *bla*_SHV_ were also tested. *E. coli* 8 strain was only *bla*_TEM_ positive and the MIC of cefquinome was 0.25 μg/mL.

### *In Vitro* Time-Killing Curves

**FIGURE 1:**
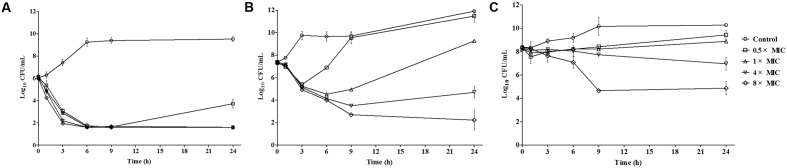
*In vitro* time-killing curves of cefquinome against *Escherichia coli* strain with different initial bacterial load. **(A)**, antibacterial effect started with 10^6^ colony counts; **(B)** bacterial density of 10^7^ CFU/mL; **(C)** initial inoculum of 10^8^ CFU/mL.

When given 10^6^ CFU/mL inoculum, bactericidal effect was observed except 0.5× MIC showing a re-growth after 9 h exposure. Re-growth phenomenon was also shown up at 0.5×, 1×, 4× MIC curves when increased the bacterial density to 10^7^ CFU/mL; as to initial inoculum of 10^8^ CFU/mL, only drug concentration of 8× MIC exhibited an over 3-log reduction of bacterial counts (**Figure 1**).

### Therapeutic Effectiveness of *E. coli* Mastitis

**FIGURE 2:**
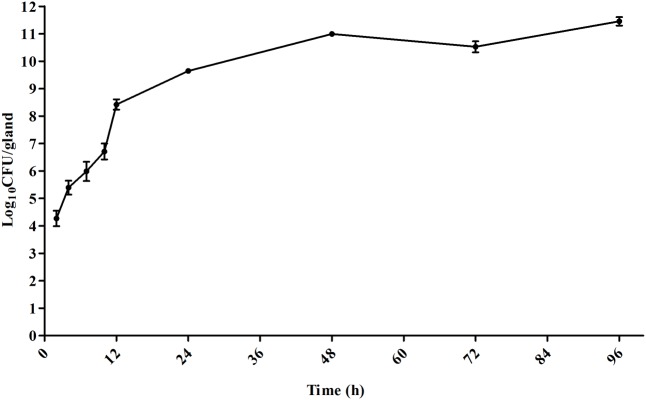
Growth curve of *E. coil* strain in mouse mammary gland tissue *in vivo* (*n* = 4 for mouse, i.e., *n* = 8 for glands).

**FIGURE 3:**
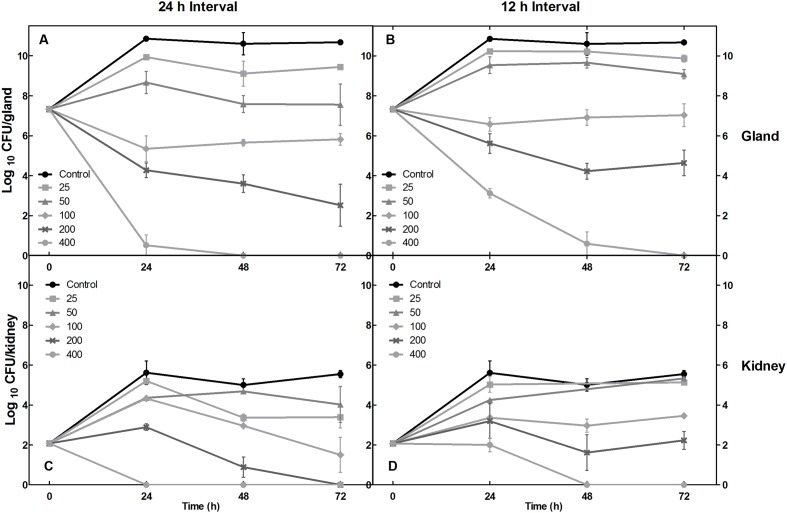
Treatment profiles of cefquinome in mammary gland and kidney tissues following an intramammary injection with daily dose ranged from 25 to 400 mg/gland for 3 days. **(A–D)** Represent the two different dosing intervals of 12 and 24 h, respectively. **(A–D)** Rows exhibit data in mammary gland and kidney organs, correspondingly.

To simulate intramammary infection in the mouse mammary gland *in vivo*, *E. coli* 8 was directly cultured in mammary tissue. A 7-log-unit target bacterial load was reached in about 10 h and by 24 and 96 h, growth was 10-log to 11-log units, respectively (**Figure 2**). The *in vivo* time-killing curves in mammary gland and kidney tissues were determined following intramammary infusion of cefquinome. The initial bacterial density was 7-log-unit per gland approximately mimicking a severe infection. Cefquinome doses equal to or less than 100 μg/gland were unable to inhibit the bacteria growth, or displayed a bacteriostatic activity with 1-log drop of cell number at most. A 7 log CFU/mL per gland killing activity which almost eliminated the infection was achieved using 400 μg/gland dosing regimens (**Figure 3**).

In mouse model after the initial inoculation, a 2-log-unit CFU was detected in kidneys. In the first 24 h, an increasing trend was observed in almost all kidney groups. When compared to mammary gland tissues, the bacterial densities in kidneys were much lower but were still maintained at 2–5 log units. In addition, similar to the findings in mammary glands, a 400 μg/gland dosage showed a therapeutic effect at 72 h, and fractional dosing caused a drop of antibacterial activity of cefquinome following intramammary administration (**Figures 3A,B**).

### Cytokine Measurements

**FIGURE 4:**
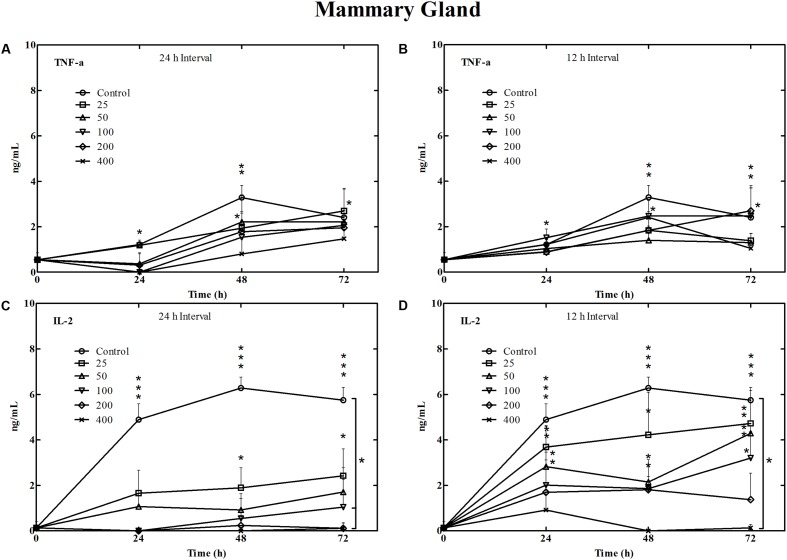
Concentrations of TNF-α **(A,B)** and IL-2 **(C,D)** cytokines in mammary gland tissue of mouse during the *E. coli* experimental infection and treatment with cefquinome for 3 days. ^∗^*P* < 0.05, ^∗∗^*P* < 0.01, and ^∗∗∗^*P* < 0.005, respectively.

During mouse *E. coli* mastitis infection, TNF-α concentrations in mammary glands of the controls and 25 μg/gland treatment groups increased significantly following bacterial challenge by 24 h (*P* < 0.05) and 48 h (*P* < 0.01). These levels remained significantly higher than the 0 h values throughout the remainder of the 72 h sampling period (*P* < 0.05). A peak TNF-α concentration of 3.29 ng/mL at 48 h in the control group contrasts with the lack of differences in the six treatment groups and between the two dosing intervals (**Figures 4A,B**).

IL-2 concentrations in mammary glands in the control and 25 μg/gland dosing groups were significantly elevated by 24 h. They remained at those levels for all 3 days at both the 12 and 24 h dosing intervals (**Figures 4C,D**). In addition, fractionated administration of 50 μg/mammary gland at 24 h and 100 μg/gland at 72 h also showed significant IL-2 increases. On the other hand, IL-2 levels in mammary glands after 400 μg/gland cefquinome were significantly lower than the no treatment group. There was also a significant difference between control mice and 200 μg/gland with 24 h dosing interval, but not with the 12 h dosing intervals (**Figures 4C,D**).

No obvious changes were observed about levels of interleukins 1, 6, and 8.

### PK, PK/PD Integration, and Modeling Analysis

The PK characteristics of mammary gland tissue were calculated by a one-compartment IV-bolus model. The *T*_1/2Kel_ was ranged from 11.56 to 13.00 h for doses of 12.5, 50, 200, and 800 μg/gland, suggesting that the drug persists at high levels in mouse glandular tissue. The *C*_0_ and AUC_0-24_ were shown in **Table 1**.

**Table 1:** Mammary gland PK profiles of cefquinome following a single intramammary infusion (mean data of six glands was used for analysis).

Dose (μg/gland)	*T*_1/2Kel_ (h)	*C*_0_ (μg/mL)	AUC_0-24_ (h⋅μg/mL)
12.5	12.95 ± 1.95	11.69 ± 0.50	292.98 ± 40.43
50	11.56 ± 0.87	35.49 ± 1.06	1059.30 ± 72.84
200	13.00 ± 1.16	146.80 ± 2.80	2753.23 ± 225.44
800	12.99 ± 1.16	587.20 ± 11.20	11004.90 ± 901.02


**T_*1/2Kel*_*_,_ half-life of elimination; *C_*0*_*, initial concentration; AUC_*0-24*_, area under the concentration-time curve.*

**FIGURE 5:**
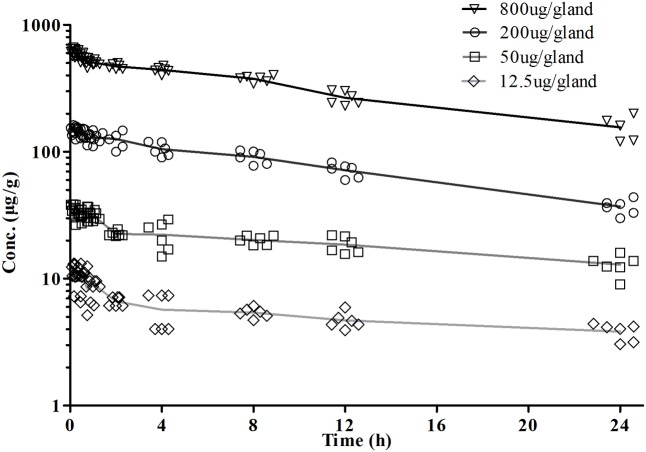
Cefquinome concentration and time plots in mammary gland tissue after a single dose of 12.5, 50, 200, and 800 μg/gland by intramammary infusion. Drug levels maintain much higher than MIC, even administrated of 12.5 μg cefquinome.

**FIGURE 6:**
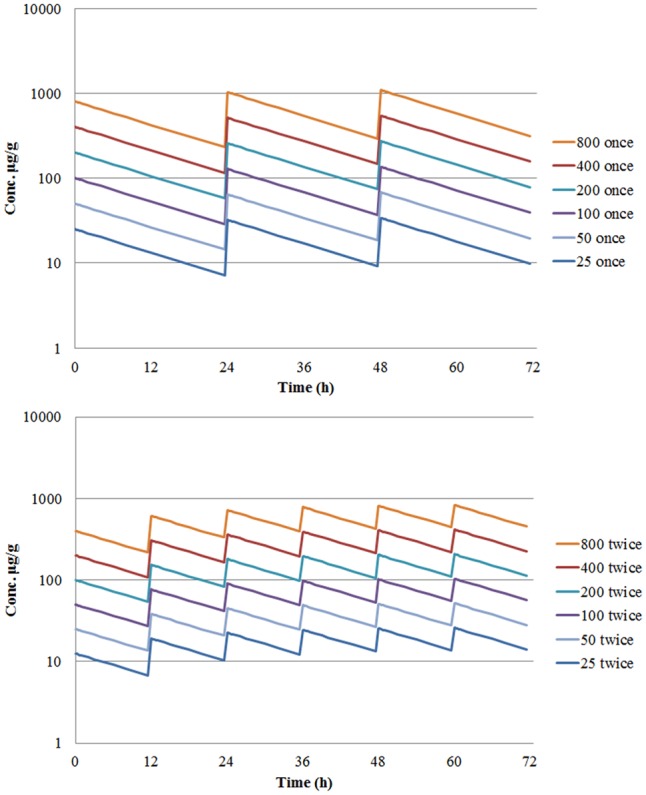
Simulation of cefquinome concentration in mammary gland tissue following multiple dosage with once daily or twice daily administration.

The concentration-time curves of cefquinome following single doses of 12.5, 50, 200, and 800 μg/gland indicated that the drug levels in mammary gland were maintained higher than MIC after 24 h (**Figure 5**). The simulation of time-concentration curves following multiple dosing were shown in **Figure 6** and *C_min_* before each administration were listed in **Table 2**. The PK/PD parameter AUC_0-24_/MIC after multiple dosing regimens were listed in **Table 3**. The drug concentrations in glandular tissues were still higher than the MIC value 24 h later after 12.5 μg/gland dosing. Therefore, the percentage duration of drug concentration exceeding the MIC value (%T > MIC) was 100% for the experimental circles.

**Table 2:** Simulating of *C_min_* (μg/g) in mammary gland tissue following multiple dosing regimens.

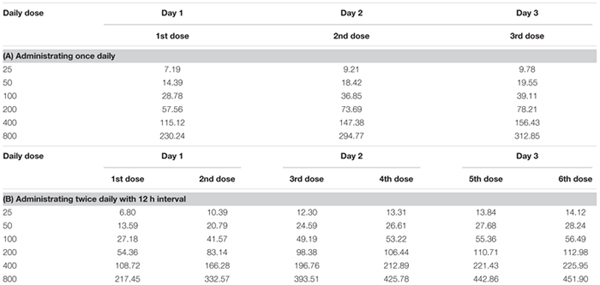

****A*** and ***B*** shows the data of once and twice daily administration, respectively.*

**Table 3:** PK/PD parameter, AUC_0-24_/MIC, of mammary gland tissue for multiple dosing regimens.

Daily Dose	Single Dose (24 h Interval)	Fractionated Dose (12 h Interval)
25	1686.55	1278.92
50	3373.10	2557.85
100	6746.20	5115.69
200	13492.40	10231.39
400	26984.79	20462.78
800	53969.58	40925.56


**FIGURE 7:**
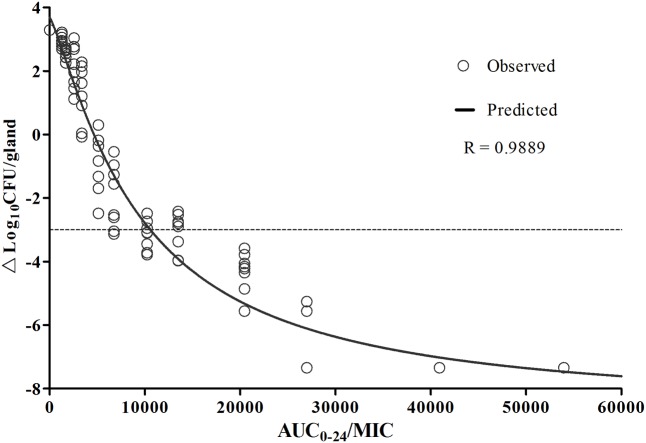
PK/PD integration of AUC/MIC *vs* antibacterial effectiveness by sigmoid *E_max_* model. The observed PD data is exhibited as circle using individual data of bacterial counts change of three mice (six glands). The predicted PD profiles were shown in line, which is calculated by Winnonlin. The dotted line represents the bactericidal effectiveness of 3-log-unit reduction.

The relationship between the PK/PD index of AUC_0-24_/MIC and cefquinome efficacy against *E. coli* 8 are shown in **Figure 7**. The correlation of observed data and predicted values was 0.9889. Analyzing by *E_max_* sigmoid model, the values of *E_max_*, *EC*_50_, and *E*_0_ for AUC_0-24_/MIC index were 3.68 ± 0.62, 9343.80 ± 2024.65, and -8.73 ± 1.30, respectively.

## Discussion

Daily cows are likely to get intramammary caused by environmental organisms infection during early lactation with obvious local and severe system clinical symptoms sometimes, which is acknowledged as environmental type of mastitis (Burvenich et al., 2003). Acute coliform mastitis is a common and usually fatal disease in lactating dairy cows (Hagiwara et al., 2014). Bacteremia has been reported to occur in 32% (Wenz et al., 2001; Cebra et al., 2010) to 75% (Katholm and Andersen, 1992) of cows with naturally occurring coliform mastitis. *E. coli* is one of the most sever cause of mastitis in the form of fatal peracute and acute with systemic reaction (Abd-Elrahman, 2013). Acute *E. coli* mastitis can be generally fatal to cows by inducing endotoxemia and disseminated intravascular coagulation (DIC) (Hagiwara et al., 2014). Endotoxemia (Katholm and Andersen, 1992), metabolic acidosis (Cebra et al., 2010), uremia (Bleul et al., 2006), and increased aspartate aminotransferase (AST) activity (Cebra et al., 2010) are commonly observed in cows with naturally occurring coliform mastitis. Overall, *E. coli* is an essential environmental pathogens inducing severe mastitis in dairy cows, which has been a considerable challenge for dairy industry worldwide.

The *E. coli* strain is capable of infecting the mouse mammary gland tissue and maintain a high bacterial density *in vivo.* Following inoculation, mental depressed, abdominal rigidity and collapsed, hair tarnished and disordered, and inactivity had been observed from infected mice in control groups. On the contrary, in treatment groups, these clinical signs were not obvious through 3 days therapy. During the therapeutic study, we found a bacterial reduction of 2-logs for fractional dosing and 3-logs for once administration at 200 μg/gland dosage at 24 h. Following three-days-therapy, a daily dose of 400 μg/gland and 200 μg/gland with 24 h intervals appeared to have a effective outcome. However, bacterial regrowth occurred when the 200 μg/gland unfractionated dose was used (**Figures 3A,B**). In kidneys, a fractionated dose of 200 μg/gland exhibited a bacteriostatic effect (no net change of colony counts) after 3 days of cefquinome treatment. This was accompanied by a 2-log reduction in the mammary gland. The blood–milk barrier may be responsible for these outcomes due to a penetration of the pathogen and an exclusion of the drug (**Figures 3A,B**) (Gruet et al., 2001; Zonca et al., 2011; Li et al., 2014).

Cytokines have the potential to improve antibacterial efficiency of certain antibiotics (Alluwaimi, 2004). In this work, we found detectable changes in IL-2 and TNF-α. The IL-2 level in mammary gland significantly elevated from healthy mice at 24 h and maintained in a high concentration until the third day of experimental circle (**Figures 4C,D**). In the contrast with non-fractionated dosing regimens, we also found much higher IL-2 levels in mammary glands with the 25 and 50 μg cefquinome/gland in the 12 h interval groups suggesting a more severe infection for these mice. It is believed that IL-2 acts to stimulate T cells to express cytokines and drives the clonal expansion and differentiation of activated T and B cells. IL-2 may be acting in such a manner in our experiments (Smith, 1988). Besides, the development of TNF-α was in accordance with the previous report that after natural E. coli mastitis it significantly increased in milk and serum (Hagiwara et al., 2000). However, the time of peak concentration in this study of 24 h for mice differed from 6 h in cows infected with lipopolysaccharide (Vels et al., 2009). It has been reported that the concentration of serum TNF-α is closely associated with acute signs and contributes to morbidity and mortality For coliform mastitis (Babiuk et al., 1991; Sordillo and Peel, 1992). TNF-α plays an essential role in immunity response by inducing plasma haptoglobin, recruiting and activating neutrophils, and elevating intramammary and systemic nitrite and nitrate (Blum et al., 2000). Previous reports have also shown that after a natural *E. coli* mastitis infection, this cytokine is significantly elevated in milk and serum (Hagiwara et al., 2000). Therefore, TNF-α in mammary gland is suggested as a candidate to monitor the severity of *coliform* mastitis (Alluwaimi, 2004).

PK profiles were similar to our previous study in lactating mice following an intramammary infusion of cefquinome (Yu et al., 2016b). The drug concentration in glandular tissue were still higher than MIC value 24 h later even given 12.5 μg/gland dosage, implying a %T > MIC maintaining for 100% just as previously reported. To explain this, tissue concentration of cefquinome and a local treatment of intramammary administration should be taken into account. In **Figure 6** and **Table 2**, drug concentration in mammary gland tissue was simulated following various dosing regimens, in which *C_min_s* before each administration were all higher than MIC value. In PK/PD modeling (**Figure 7**), the tail of simulation curve leveled off, so it was not necessary to use the highest dosage. The break point of the sigmoid *E_max_* curve was located near AUC_0-24_/MIC of 10,000 ∼ 20,000 (**Figure 7**). To define the best magnitude of AUC_0-24_/MIC, not only PK/PD integration but also the therapeutic effectiveness and cytokines data have been taken into consideration. According to these outcomes, the AUC_0-24_/MIC of 13492.40 h, following an once infusion of 200 μg/gland cefquinome, was defined as the target magnitude of PK/PD index.

For the consideration of animal bulk, size of glandular/udder, volume of milk, etc., it may be inappropriate to transform the dose of 200 μg/gland from mouse to cows, or improper to compare these two regimens, directly. Although non-target animal studies are not able to directly define the optimal clinical dose regimen when considering the species difference, they are still capable of defining the magnitude of the PK/PD index required for different treatment outcomes since various animal species including human should share a similar magnitude of the PK/PD index (Craig, 1998; Andes and Craig, 2002). In previous report, AUC_last_ of milk concentration was determined to be about 1592.99 h⋅μg/mL following 75 mg cefquinome infusion in health quarter, and MICs against 637 *E. coli* strains were ranged from 0.06 to 0.13 μg/mL (Zonca et al., 2011). Based on these data, the AUC_last_/MIC was ranged from 13266.7 to 26533.33 approximately, which is comparable to the magnitude of AUC_0-24_/MIC in the current study. In addition, drug concentration in different tissues samples have critical influence on the AUC/MIC value. For instance, drug concentration in milk samples could be lower than in glandular tissue due to dilution effect (Zonca et al., 2011). On the contrary, drug level could be overvalued in homogenate tissue than the real situation, because cefquinome can barely cross through cell membrane.

In summary, this is the first study ever to assess glandular tissue PK/PD integration for investigating the effectiveness of cefquinome against environmental *E. coli* mastitis. In this work, environmental *E. coli* pathogen was identified by phylogenetic typing and tested for antibiotic resistant genes. During the 3 days treatment, *in vivo* therapeutic efficacy and expression of cytokines were also detected. In addition, the PK/PD modeling were performed based on the mammary gland tissue PK data and PD outcomes in local glandular organs. Above all, the magnitude of PK/PD parameters to achieve a remarkable antibacterial efficacy is evaluated in this study, considering the facts of PD activity, expression of cytokines, and PK/PD modeling.

The advantages of a local treatment and a parenteral one are still controversial and discussed. To achieve a reliable curative effect, it is better to choose regimens based on the clinical symptoms. Local treatment may be recommended for using in preliminary stage of infection. On the contrary, when systemic infection has been developed, a parenteral administration (with intramammary infusion or not) might be a wise choice. In addition, it is well known that not only bacteria but also environment, management, and cows themselves are all factors responsible for mastitis (Burvenich et al., 2003). Therefore, antibacterial movements, by applying antibiotics is insufficient for preventing environmental mastitis, more strategies are urgently required with respects to reinforcing the farming circumstances, scientific managements and improving host immunity.

## Ethics Statement

This study was carried out in accordance with the recommendations of the guidelines of American Association for Accreditation of Laboratory Animal Care. The protocol was approved by the Animal Use and Care Committee of South China Agricultural University.

## Author Contributions

Y-HL conceived of the study and given the final approval of the version to be published. YY participated in design of the study and drafted the manuscript. J-TF and J-SH carried out the pharmacokinetic studies and animal experiments of pharmacodynamic work. X-PL have made substantial contribution to analysis and interpretation of data. JS, MZ, and QZ has been involved in revising the manuscript critically for important intellectual content. All authors read and approved the final manuscript.

## Conflict of Interest Statement

The authors declare that the research was conducted in the absence of any commercial or financial relationships that could be construed as a potential conflict of interest.
